# Supplementary material for: FACT depletion demonstrates a role for nucleosome organization in TAD formation
Source: Mol Syst Biol. 2025 Oct 27;22(1):8. doi: 10.1038/s44320-025-00165-7 (PMC12759068; doi:10.1038/s44320-025-00165-7)
Supplement: Supplementary file 2 — Expanded View Figures/Table [file 44320_2025_165_MOESM2_ESM.pdf]

# Expanded View Figures/Table

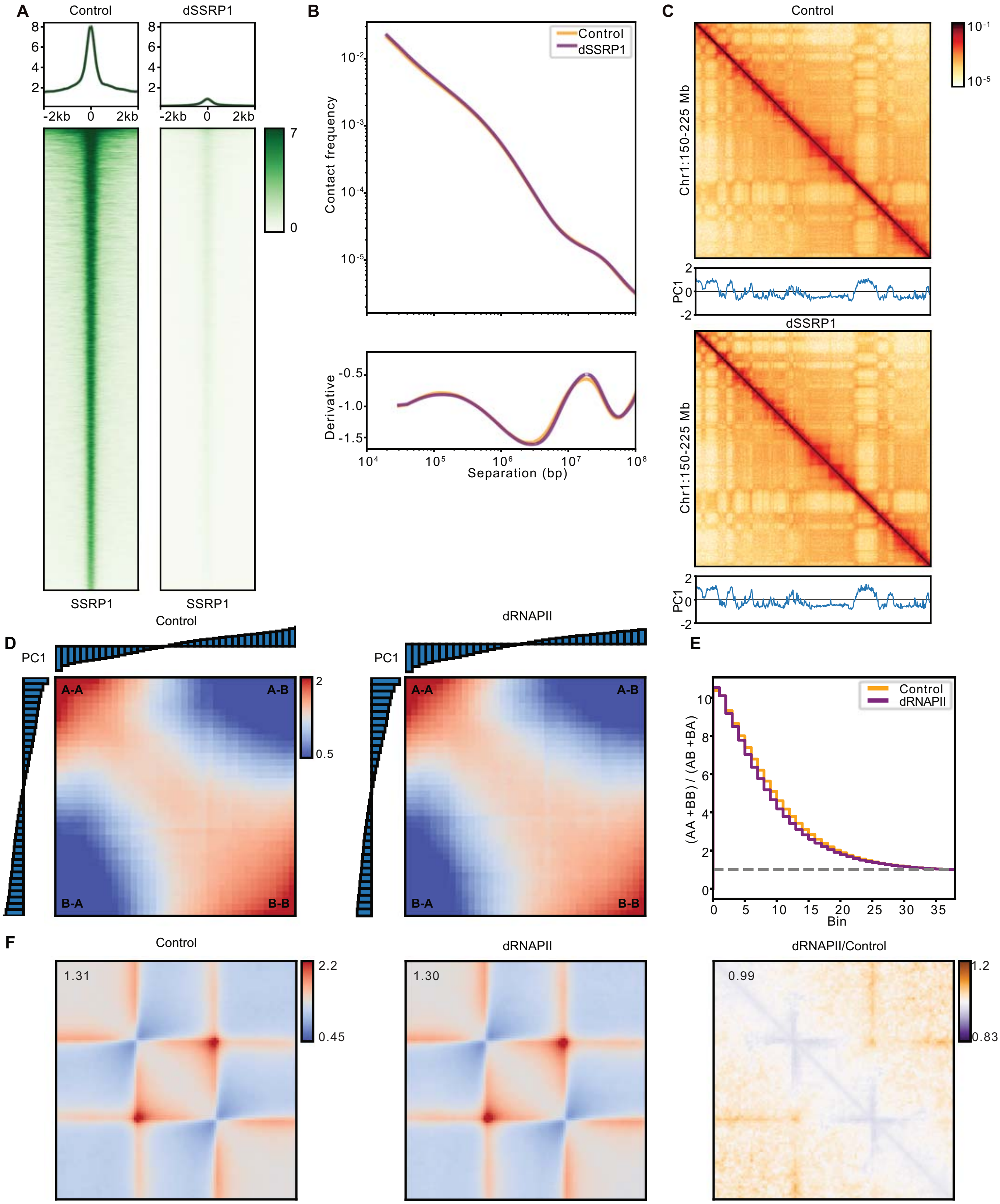

**Figure EV1. Rapid depletion of FACT leads to changes in 3D genome organization that are not secondary to changes in transcription.**

(**A**) SSRP1 ChIPmentation signal in control (left) and SSRP1-depleted (right) K562 cells. ChIPmentation data show the average of 2 biological replicates. (**B**) Micro-C contact frequency decay as a function of genomic distance (top) and its first derivative (bottom) in control (orange) and SSRP1-depleted (purple) cells. Data as in Fig. 1A. (**C**) Micro-C contact matrices (100 kb resolution) of an exemplary region on chromosome 1 in control (left) and SSRP1-depleted (right) cells. Below is the Eigenvector indicating A compartment ( > 0) or B compartment ( < 0). Data as in Fig. 1A. (**D**) Saddleplots displaying observed/expected intra- and intercompartment interactions of genomic regions stratified into 39 bins by Eigenvector decomposition in control (left) and RNAPII-depleted cells (right). Histograms show Eigenvalues of stratified bins. Micro-C data show the average of 2 biological replicates. (**E**) Step-plot showing the saddle strength profile (intracompartmental/intercompartmental interactions) in control (orange) and RNAPII-depleted (purple) cells. Data as in (**D**). (**F**) Mean observed/expected contact frequencies at TADs ($n = 2831$) in control (left) and RNAPII-depleted (middle) cells and their relative differences (right; purple indicates enriched contacts in control cells; orange indicates enriched contacts in RNAPII-depleted cells). TADs are rescaled to the same size. Average signal is reported in the top left. Data as in (**D**).

 © The Author(s)

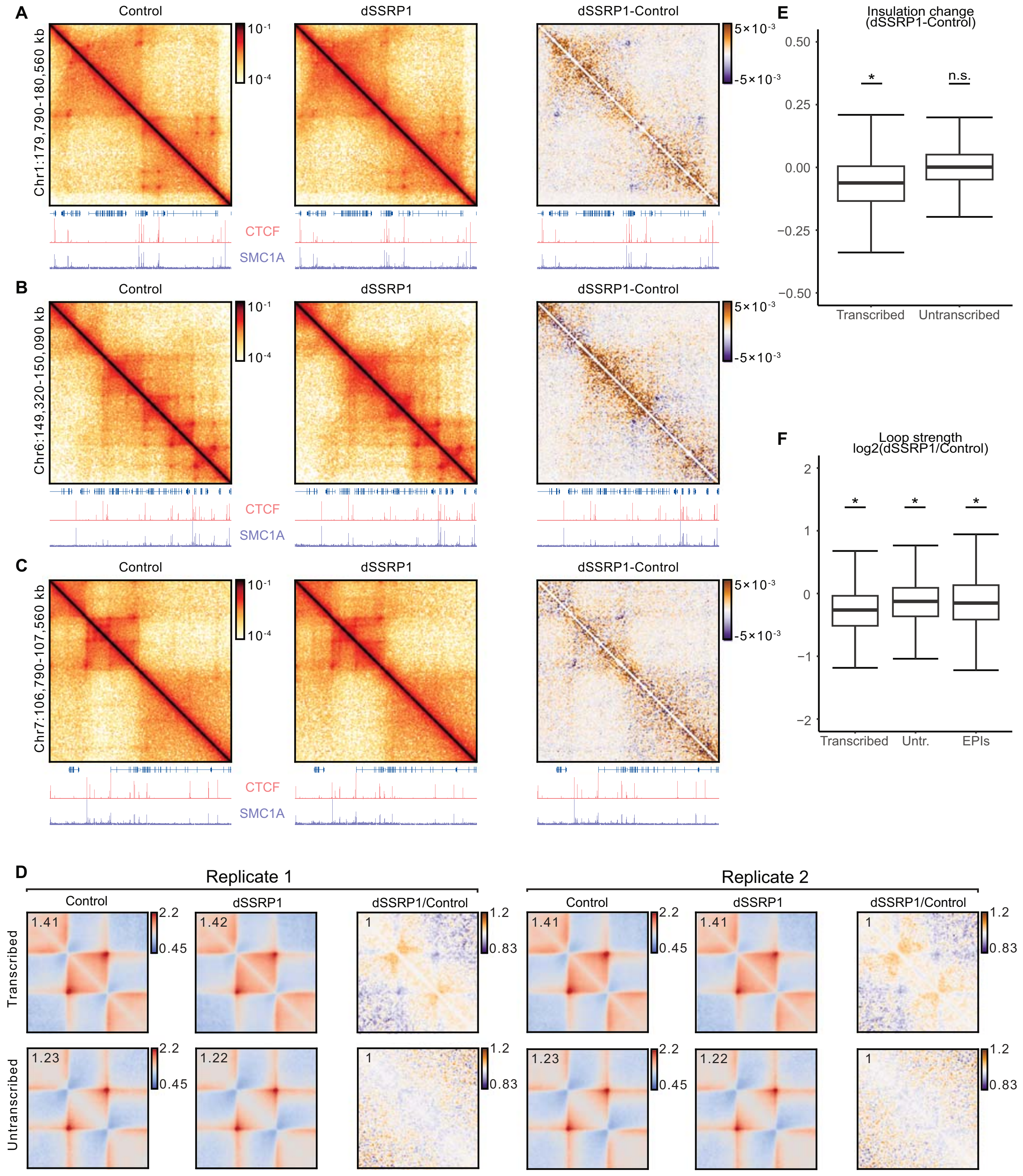
A
Control
dSSRP1
dSSRP1-Control
Chr1:179,790-180,560 kb
CTCF
SMC1A
B
Chr6:149,320-150,090 kb
C
Chr7:106,790-107,560 kb
D
Replicate 1
Replicate 2
dSSRP1/Control
Transcribed
Untranscribed
E
Insulation change
(dSSRP1-Control)
n.s.
Untranscribed
F
Loop strength
log2(dSSRP1/Control)
Untr.
EPIs

**Figure EV2. Rapid depletion of FACT leads to significant changes in 3D genome organization that are consistent across genomic regions and replicates.**

(**A**) Micro-C contact matrices (5 kb resolution) of an exemplary region on chromosome 1 in control (left) and SSRP1-depleted (middle) K562 cells and their absolute differences (right; purple indicates enriched contacts in control cells; orange indicates enriched contacts in SSRP1-depleted cells). Gene annotation and ChIPmentation tracks for CTCF and SMC1A in control (left and right) and SSRP1-depleted cells (middle) are shown at the bottom. Data as in Fig. 2A. (**B**) Same as (**A**) for an exemplary region on chromosome 6. (**C**) Same as (**A**) for an exemplary region on chromosome 7. (**D**) Mean observed/expected contact frequencies at transcribed (top) and untranscribed (bottom) TADs in independent replicates of control (left) and SSRP1-depleted (middle) cells and their relative differences (right; purple indicates enriched contacts in control cells; orange indicates enriched contacts in SSRP1-depleted cells). TADs are rescaled to the same size. Average signal is reported in the top left. Data as in Fig. 2A. (**E**) Relative differences in insulation scores at transcribed (left; two-sided Wilcoxon rank-sum test; $n = 1811$, $P < 2.2e\text{-}16$) and untranscribed (right; two-sided Wilcoxon rank-sum test; $n = 1948$, $P < 2.2e\text{-}16$) TAD boundaries between control and dSSRP1-depleted cells. Boxplots show the interquartile range (IQR) and median of the data; whiskers indicate the minima and maxima within 1.5 * IQR; asterisks indicate statistical significance ($P < 0.05$); n.s. = non-significant. Data as in Fig. 2A. (**F**) Same as (**E**) for CTCF loop strength at transcribed (left; two-sided Wilcoxon rank-sum test; $n = 657$, $P < 2.2e\text{-}16$) and untranscribed (Untr.; middle; two-sided Wilcoxon rank-sum test; $n = 900$, $P < 2.2e\text{-}16$) TAD boundaries and enhancer–promoter interactions (right; two-sided Wilcoxon rank-sum test; $n = 423$, $P < 9.718e\text{-}11$).

 © The Author(s)

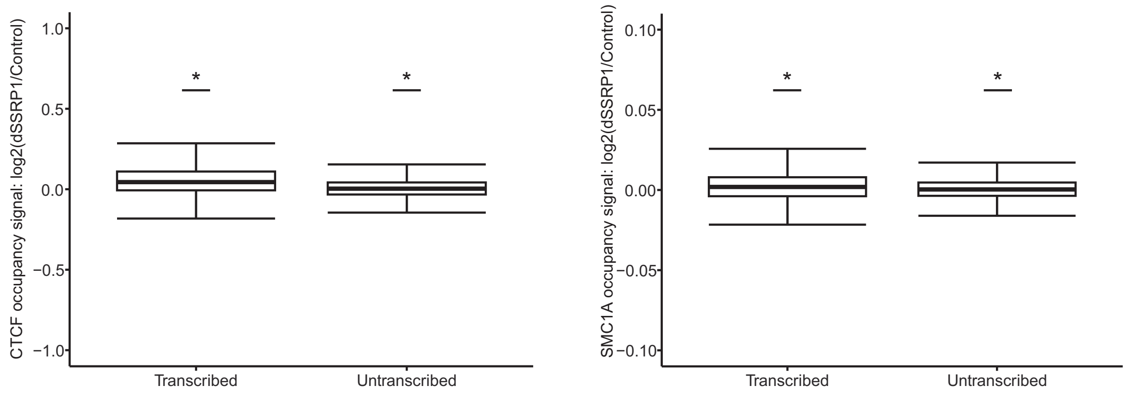


**Figure EV3. The effects of FACT depletion on TAD organization are not dependent on reduced CTCF and cohesin occupancy.**

Relative differences in CTCF (left panel) and SMC1A (right panel) occupancy between control and dSSRP1-depleted K562 cells in transcribed (left box; two-sided Wilcoxon rank-sum test; $n = 22{,}681$, $P < 2.2e\text{-}16$) and untranscribed (right box; two-sided Wilcoxon rank-sum test; $n = 20{,}099$, $P < 2.2e\text{-}16$) regions. Boxplots show the interquartile range (IQR) and median of the data; whiskers indicate the minima and maxima within 1.5 * IQR; asterisks indicate statistical significance ($P < 0.05$). Data as in Fig. 3.

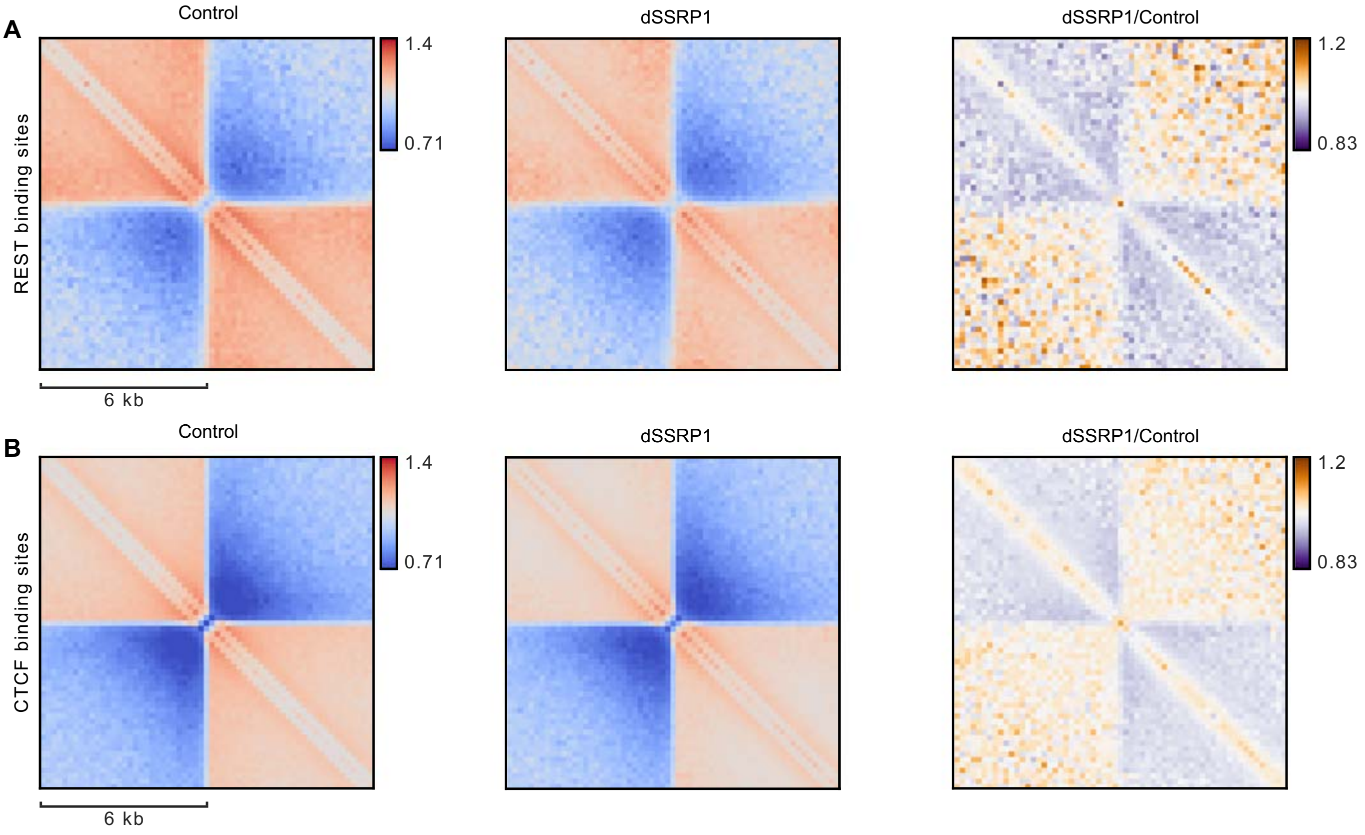


**Figure EV4. Local insulation at REST and CTCF binding sites.**

(**A**) Mean observed/expected contact frequencies at REST binding sites (that do not overlap with CTCF binding sites; $n = 13{,}387$) in control (left) and SSRP1-depleted (middle) K562 cells and their relative differences (right; purple indicates enriched contacts in control cells; orange indicates enriched contacts in SSRP1-depleted cells). Note that insulation shown here extends over much shorter distances compared to insulation at CTCF-bound TAD borders shown in Fig. 1C. Data as in Fig. 2A. (**B**) Same as (**A**) at all CTCF binding sites across the genome (including CTCF binding sites that do not overlap with TAD borders; $n = 57{,}154$).

**Table EV1. Micro-C mapping, deduplication, and contact statistics from 2 merged biological replicates.**

| | Control | | dSSRP1 | |
|---|---|---|---|---|
| | Read number | Percentage | Read number | Percentage |
| Total read pairs | 2,033,336,706 | 100 | 1,830,972,576 | 100 |
| Mapped read pairs | 1,821,115,104 | 89.6 | 1,626,027,133 | 88.8 |
| Duplicate read pairs | 304,308,463 | 26.6 | 260,134,033 | 25.8 |
| Valid *trans* read pairs | 152,853,931 | 13.4 | 127,161,826 | 12.6 |
| Valid *cis* read pairs | 686,790,761 | 60 | 620,232,513 | 61.6 |

Percentages in top two rows are relative to total number of read pairs; percentages in bottom three rows are relative to total number of uniquely aligned read pairs.

 © The Author(s)
